# Unveiling the role of the KLF4/Lnc18q22.2/ULBP3 axis in the tumorigenesis and immune escape of hepatocellular carcinoma under hypoxic condition

**DOI:** 10.1111/jcmm.18411

**Published:** 2024-05-23

**Authors:** Lifang Wei, Ping He, Zhongqiu Tan, Lifeng Zhao, Cheng Lin, Zhongheng Wei

**Affiliations:** ^1^ Health Management Center The Affiliated Hospital of Youjiang Medical University for Nationalities Baise Guangxi China; ^2^ School of Laboratory Medicine Youjiang Medical University for Nationalities Baise Guangxi China; ^3^ Department of Oncology The Affiliated Hospital of Youjiang Medical University for Nationalities Baise Guangxi China; ^4^ Guangxi Clinical Medical Research Center for Hepatobiliary Diseases The Affiliated Hospital of Youjiang Medical University for Nationalities Baise China

**Keywords:** hepatocellular carcinoma, hypoxia, immune escape, KLF4, Lnc18q22.2, tumorigenesis, ULBP3

## Abstract

Hepatocellular carcinoma (HCC) represents a significant global health burden, necessitating an in‐depth exploration of its molecular underpinnings to facilitate the development of effective therapeutic strategies. This investigation delves into the complex role of long non‐coding RNAs (lncRNAs) in the modulation of hypoxia‐induced HCC progression, with a specific emphasis on delineating and functionally characterizing the novel KLF4/Lnc18q22.2/ULBP3 axis. To elucidate the effects of hypoxic conditions on HCC cells, we established in vitro models under both normoxic and hypoxic environments, followed by lncRNA microarray analyses. Among the lncRNAs identified, Lnc18q22.2 was found to be significantly upregulated in HCC cells subjected to hypoxia. Subsequent investigations affirmed the oncogenic role of Lnc18q22.2, highlighting its critical function in augmenting HCC cell proliferation and migration. Further examination disclosed that Kruppel‐like factor 4 (KLF4) transcriptionally governs Lnc18q22.2 expression in HCC cells, particularly under hypoxic stress. KLF4 subsequently enhances the tumorigenic capabilities of HCC cells through the modulation of Lnc18q22.2 expression. Advancing downstream in the molecular cascade, our study elucidates a novel interaction between Lnc18q22.2 and UL16‐binding protein 3 (ULBP3), culminating in the stabilization of ULBP3 protein expression. Notably, ULBP3 was identified as a pivotal element, exerting dual functions by facilitating HCC tumorigenesis and mitigating immune evasion in hypoxia‐exposed HCC cells. The comprehensive insights gained from our research delineate a hitherto unidentified KLF4/Lnc18q22.2/ULBP3 axis integral to the understanding of HCC tumorigenesis and immune escape under hypoxic conditions. This newly unveiled molecular pathway not only enriches our understanding of hypoxia‐induced HCC progression but also presents novel avenues for therapeutic intervention.

## 1 INTRODUCTION

Liver cancer exhibited a significant global impact, ranking it as the sixth most diagnosed cancer and the third leading cause of cancer‐related deaths worldwide in the year 2020.^1^ Hepatocellular carcinoma (HCC) constitutes the predominant form, making up 85%–95% of primary liver cancer cases. Unfortunately, about 80% of individuals with HCC receive a diagnosis at advanced stages, missing the window for surgical interventions. Consequently, the overall 5‐year survival rate remains below 30%, accompanied by an 80% recurrence rate in advanced HCC patients.^2^, ^3^ Consequently, there is an urgent need to unravel the mechanisms that drive HCC progression.

Tumours represent heterogeneous diseases, and the majority of solid tumours, as observed in most cases, exhibit hypoxia in comparison to normal tissues.^4^ As cancer progresses, abnormal neovascularization fails to adequately maintain blood supply, resulting in chronic tumour hypoxia.^5^ Low primary tumour oxygenation is correlated with an elevated risk of metastasis and increased patient mortality.^6^ To adapt and survive under hypoxic conditions, tumour cells undergo transcriptional alterations in numerous genes and activate multiple oncogenic signalling pathways, thereby orchestrating a malignant cellular phenotype.^7^ Tumour hypoxia responses are primarily mediated by hypoxia‐inducible factors (HIFs), governing processes such as cell growth, angiogenesis, metabolism and glycolysis.^8^, ^9^ Nevertheless, the intricate molecular mechanisms driving hypoxia‐induced HCC progression remain predominantly unclear, warranting further studies for a comprehensive understanding.

Even though long non‐coding RNAs (lncRNAs) lack the ability to encode proteins, their crucial roles in various cellular activities should not be underestimated. A growing body of evidence suggests that lncRNAs play pivotal roles in various processes within HCC cells.^10^, ^11^, ^12^, ^13^, ^14^, ^15^, ^16^

In the current study, we aimed to rigorously investigate the influence of lncRNAs on the progression of HCC under hypoxic conditions. To achieve this, we developed HCC cell models subjected to both normoxic and hypoxic environments, followed by a comprehensive lncRNA microarray analysis. This analysis led to the identification of Lnc18q22.2 as the lncRNA most significantly upregulated in response to hypoxic stress in HCC cells. Subsequent functional characterization of Lnc18q22.2 established its oncogenic role in the context of hypoxia‐induced HCC progression, notably through its promotion of cellular proliferation and migration. Further exploration revealed the transcriptional regulation of Lnc18q22.2 by Kruppel‐like factor 4 (KLF4) within HCC cells. Notably, under hypoxic conditions, KLF4 was found to enhance the tumorigenic properties of HCC cells by modulating Lnc18q22.2 expression. Our investigation extended to the downstream effects of Lnc18q22.2, uncovering its interaction with and stabilization of UL16‐binding protein 3 (ULBP3) protein expression. Intriguingly, ULBP3 was shown to play a dual role, not only promoting HCC tumorigenesis but also inhibiting immune escape mechanisms in HCC cells under hypoxic conditions.

The collective results of our study elucidate a previously unidentified KLF4/Lnc18q22.2/ULBP3 axis integral to HCC tumorigenesis and the evasion of immune surveillance under hypoxic conditions. This discovery offers novel insights into the mechanisms driving hypoxia‐induced HCC progression and opens new avenues for therapeutic intervention targeting these specific molecular interactions.

## 2 MATERIALS AND METHODS

### 2.1 Cell culture and treatment

This study utilized various human HCC cell lines, namely SMMC‐7721 and Hep3B. Cells were sourced from the Chinese Academy of Sciences (CAS). SMMC‐7721 cells were cultured in RPMI 1640 medium (61870127, Gibco, USA) supplemented with 10% fetal bovine serum (FBS; 10270‐106, Gibco) and 1% penicillin–streptomycin solution (SV30010, PERFEMIKER, Shanghai, China). Hep3B cells were cultured in DMEM (12100046, Gibco) with the same supplements as mentioned earlier. All incubators were maintained in a humidified environment with 5% CO_2_ at 37 °C. To induce hypoxia in the HCC cells, they were either kept in an incubator with 5% CO_2_, 1% O_2_, and 94% N_2_ or treated with 100 μM CoCl_2_ for 24 h. For cell transfection, Initially, HCC cells were seeded into six‐well plates and allowed to incubate overnight. Subsequently, the cells were harvested, and various plasmids were introduced for RNA knockdown or overexpression purposes. Short hairpin RNAs and pcDNA3.1 were synthesized and applied. These designed plasmids, alongside a negative control (NC), were then transfected into HCC cells utilizing Lipofectamine 2000 (11668019, Invitrogen, USA).

### 2.2 lncRNA microarray analysis

Total RNA was extracted from cells exposed to lipopolysaccharide (LPS) using Trizol reagent (Invitrogen, Carlsbad, CA), and its concentration was determined with the NanoDrop ND‐1000 (Thermo Scientific, Wilmington, DE). Kangchen Corporation (Shanghai, China) conducted lncRNA microarray analysis following Arraystar's standard procedures. In a nutshell, RNA samples underwent amplification, transcription, hybridization, and labeling according to the Arraystar lncRNA Array (6x7K; Arraystar Inc., Rockville, MD) protocol. The microarray was subsequently analysed using the Arraystar lncRNA Microarray and scanned with the Agilent G2505C Scanner.

### 2.3 qRT‐PCR

Total RNA from HCC cells was extracted using TRIzol (9108‐1, Thermo Fisher, USA). Subsequently, the obtained RNA underwent reverse transcription to generate complementary DNA (cDNA). The expression levels of the RNAs were then quantified using a qRT–PCR kit (QR0100‐1KT, Sigma–Aldrich, USA). The primer sequences information is listed as following: Lnc18q22.2 FOR 5’‐GGAGGCTGTTGACAGGCAATG‐3′; REV 5′‐GACTGCAACTTAAGCTATCTGG‐3′. KLF4 FOR 5’‐TCCCATCTTTCTCCACGTTC‐3′; REV 5’‐AGTCGCTTCATGTGGGAGAG‐3′. ULBP3 FOR 5′‐CTGGAACTGGCTGACACTGA‐3′; REV 5′‐TGGTCAGTCCGCTATCCTTC‐3′. GAPDH FOR 5′‐AGCCCAAGATGCCCTTCAGT‐3′; REV 5′‐CCGTGTTCCTACCCCCAATG‐3′. U6 FOR 5′‐CGCTTCGGCAGCACATATAC‐3′; REV 5′‐CAGGGGCCATGCTAATCTT‐3′. The 2^−∆∆Ct^ method was applied for result quantification, with GAPDH serving as the internal reference.

### 2.4 Western blot

Protein extraction from HCC cells was conducted using protein lysis buffer (ZD409, ZOMANBIO, Beijing, China) and a Total Protein Extraction Kit (BC3711, Solarbio, Beijing, China). The obtained proteins were separated via SDS–PAGE (P1200, Solarbio) and then transferred onto PVDF membranes (T2234, Thermo Fisher). Following blocking with 5% skimmed milk, the membranes were incubated with primary antibodies. Following this, the membranes underwent incubation with secondary antibodies for 1 h. Protein levels were determined using an enhanced chemiluminescence detection system, with β‐actin serving as the internal reference.

### 2.5 CCK‐8

Cell viability was evaluated using a CCK‐8 assay (Dojindo, Japan). Following a 48‐h treatment, HCC cells were harvested, and 2 × 10^3^ HCC cells were seeded in 96‐well plates for further incubation. Subsequently, 90 μL of complete medium with 10 μL of CCK‐8 reagent was added to each well and incubated for 2 h. The absorbance was then measured at 450 nm.

### 2.6 Cell colony

The specified cells (500 cells/3 mL) were seeded into 12‐well plates and incubated for 8–12 days under conditions normoxia and hypoxia, with media changes every 3 days. After the incubation period, the cell colonies were rinsed once with PBS and fixed with 0.5% paraformaldehyde for 20 min. Following fixation, the colonies were stained with crystal violet and subsequently counted. All experiments were replicated three times, yielding consistent results.

### 2.7 Transwell migration

To evaluate the migration ability of PC cells, a transwell migration assay was employed. In brief, transwell chambers were coated with 6 × 10^4^ HCC cells in 200 μL of serum‐free medium, while the lower chambers contained 700 μL of complete medium. After 24 h of culture, the upper chambers, housing the migrated HCC cells, were fixed, and stained with a 1% crystal violet stain solution for 20 min. Subsequently, the migrated HCC cells were manually counted under a microscope. The relative proportions of migrated cells were calculated as the percentages of treated cells compared to the NC cells.

### 2.8 Chromatin immunoprecipitation (ChIP)

To carry out the ChIP assay, a ChIP Kit (KT101‐01, gzscbio, Guangzhou, China) was acquired. In brief, crosslinked chromatin was fragmented into small sections. Subsequently, a mixture of antibodies, along with magnetic beads, was added for co‐incubation. Finally, qPCR was performed to assess the expression of enriched DNA.

### 2.9 RNA binding protein immunoprecipitation (RIP) assay

The RIP assay was conducted using an Imprint® RNA Immunoprecipitation Kit (RIP‐12RXN, Sigma–Aldrich, USA), strictly adhering to the manufacturer's instructions. Cell lysates were incubated with magnetic beads conjugated with NC anti‐immunoglobulin G (IgG) (ab6789, Abcam, USA), anti‐ULBP3. Following purification, the immunoprecipitated RNAs were isolated, and quantitative data were acquired through RT–qPCR analyses.

### 2.10 RNA pull‐down and LC–MS/MS

Lnc18q22.2 and the control were transcribed in vitro, supplemented with U‐biotin using an RNA transcription kit (#P1320, Promega) from linearized constructs, and then purified with TRIzol reagent (Invitrogen). The in vitro transcribed RNAs were incubated in RNA structure buffer (10 mM Tris–HCl, pH = 7; 0.1 M KCl; and 10 mM MgCl2) and heated to 72°C for 2 min to form the appropriate secondary structures. Subsequently, these RNAs were incubated with cell lysates (10^8^ cells cultured under hypoxia for 12 h) at 4°C for 4 h, followed by incubation with streptavidin beads (Invitrogen) at room temperature for 1 h. After five washes, the pulldown complexes were analysed either by LC–MS/MS identification or western blotting.

### 2.11 Luciferase reporter gene assay

The Lnc18q22.2 promoter sequences, both wild‐type (WT) and sites‐mutated, were cloned into pGL3 reporter vectors to generate pGL3‐Lnc18q22.2 promoter (WT) and pGL3‐Lnc18q22.2 promoter (Sites Mut). Following plasmid transfection, a Dual Luciferase Reporter Assay Kit (DL101‐01, Vazyme, Nanjing, China) was employed to assess luciferase activities according to the manufacturer's instructions. After a 48 h transfection period, the relative luciferase activities were measured and normalized to Renilla reniformis activity.

### 2.12 NK cell cytotoxicity detection

The assessment of NK cell cytotoxicity involved detecting the level of lactate dehydrogenase (LDH) using the CytoTox96 Non‐Radioactive Cytotoxicity Assay Kit (Promega, Madison, WI, USA). In brief, cells were plated at a concentration of 2 × 10^5^ cells/mL into a 96‐well plate. Subsequently, 50 μL of CytoTox96 Reagent was added to each well and incubated for 30 min at room temperature, followed by the addition of Stop Solution (50 μL). The absorbance at 490 nm (A490) was measured using a microplate reader (Bio‐Rad). Percent cytotoxicity was calculated according to the kit instructions.

### 2.13 ELISA

The levels of IFN‐γ and TNF‐α in the cell culture medium were quantified utilizing ELISA kits (BMS228 and BMS223‐4, Invitrogen). In summary, the culture medium was collected and centrifuged at 1400 rpm for 1 min. The ELISA assay was conducted according to the provided instructions, and the absorbance at 450 nm (A450) was measured using a microplate reader (Bio‐Rad).

### 2.14 Statistical analysis

Statistical analyses were performed using the SPSS software package and GraphPad Prism. Each independent experiment was conducted a minimum of three times. The data are expressed as the mean ± standard deviation (SD). Differences between groups were assessed using Student's t‐test, one‐way analysis of variance (ANOVA), or two‐way ANOVA. A significance level of *p* < 0.05 was considered statistically significant.

## 3 RESULTS

### 3.1 Lnc18q22.2 is upregulated in hypoxia induced HCC cells

**FIGURE 1:**
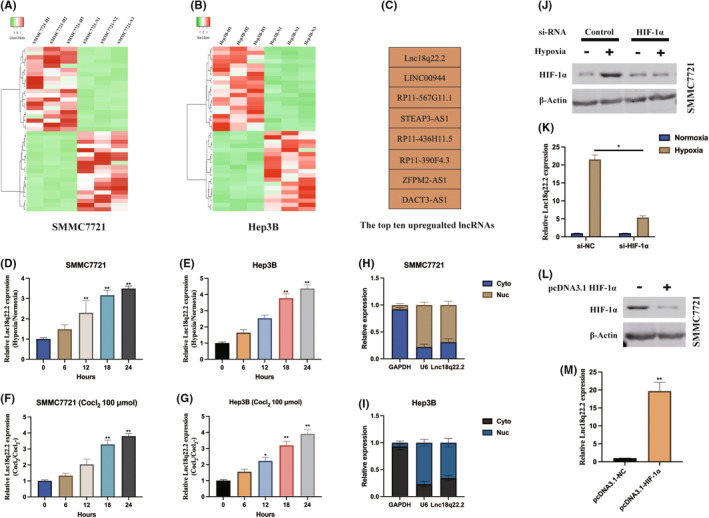
Lnc18q22.2 is upregulated in hypoxia induced HCC cells. (A, B) SMMC7721 and Hep3B cells were cultured under normoxic and hypoxic conditions and then subjected to lncRNA microarray analysis. (C) The top eight significantly upregulated lncRNAs were listed as indicated. (D, E) HCC cells were subjected to treatment under hypoxic/normoxic, Lnc18q22.2 was measured at different time poinst as indicated. (F, G) HCC cells were exposed to 100 μM CoCl2 with different concentrations 8 h, Lnc18q22.2 was measured as indicated. (H, I) the subcellular distribution of Lnc18q22.2 in HCC cells was validated. (J, K) SMMC7721 cells underwent transfection with si‐NC and si‐HIF‐1α. Following a 24‐h transfection period, cells were cultured under either normoxic or hypoxic conditions for an additional 24 h. The expression levels of HIF‐1α were assessed through western blot analysis, while the levels of Lnc18q22.2 expression were determined using qRT‐PCR. (L, M) SMMC7721 cells were transfected with pcDNA3.1‐HIF‐1α. After a 24‐h transfection period, the expression level of HIF‐1α was assessed through western blot analysis. Concurrently, the levels of Lnc18q22.2 expression were examined using qRT‐PCR. The results were presented as mean ± SD with a sample size of *n* = 3.

To explore lncRNAs associated with hypoxia in HCC, we analysed lncRNA expression profiles in SMMC7721 and Hep3B cells under normoxic and hypoxic (1% O_2_, 48 h) conditions (Figure 1A,B). The top eight upregulated lncRNAs are presented in Figure 1C. Subsequently, we observed a time‐dependent increase in Lnc18q22.2 expression in HCC cells subjected to hypoxic/normoxic treatment (Figure 1D,E). Moreover, Lnc18q22.2 was significantly upregulated in response to cobalt chloride treatment, a hypoxia‐mimetic agent, in a dose‐dependent manner (Figure 1F,G). The subcellular distribution of Lnc18q22.2 was investigated, revealing its predominant localization in the cytoplasm of SMMC7721 and Hep3B cells. Additionally, we found that HIF‐1α positively regulates Lnc18q22.2 expression in SMMC7721 and Hep3B cells under hypoxia (Figure 1J–M).

### 3.2 Lnc18q22.2 contributes to HCC tumorigenic properties under hypoxia

**FIGURE 2:**
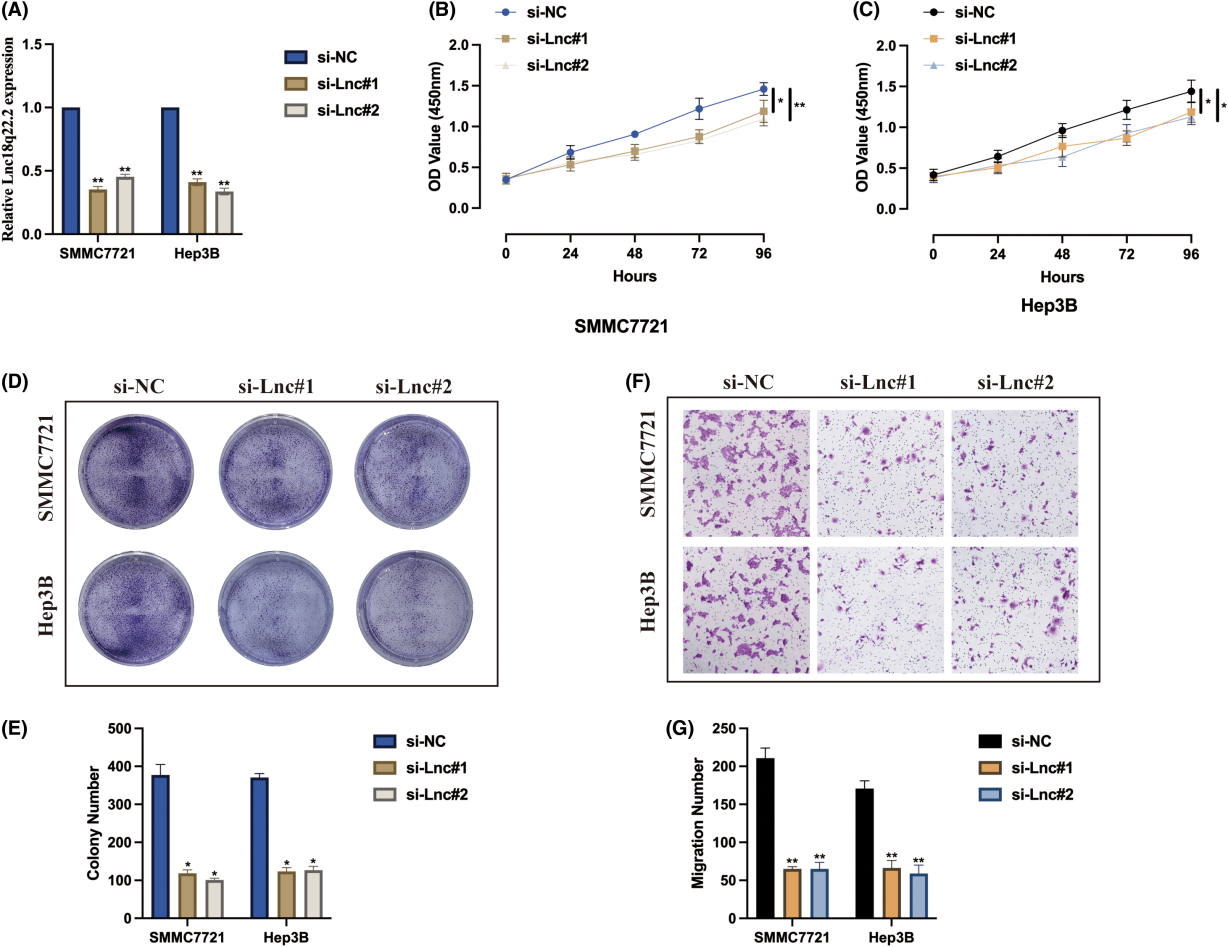
Lnc18q22.2 contributes to HCC tumorigenic properties under hypoxia. (A) SMMC7721 and Hep3B cells were transfected with si‐NC, si‐Lnc#1 and si‐Lnc#2. Following a 24‐h transfection period, cells were cultured under either normoxic or hypoxic conditions for an additional 24 h. The levels of Lnc18q22.2 expression were examined using qRT‐PCR. (B, C) Cell proliferation levels were determined by CCK‐8 assay. (D, E) Cell viability was detected by cell colony assay. (F, G) Cell migration ability was assessed by transwell migration assay. The results were presented as mean ± SD with a sample size of *n* = 3.

To understand the biological role of Lnc18q22.2, we stably generated Lnc18q22.2 knockdown cell models under hypoxia condition, transfection efficiencies were validated as indicated in Figure 2A. The influence of Lnc18q22.2 on hypoxia induced HCC cell proliferation were verified. As demonstrated by CCK‐8 assay, Lnc18q22.2 knockdown significantly inhibited HCC cell proliferation level under hypoxia condition (Figure 2B,C). The same phenomena were also observed by utilizing cell colony assay (Figure 2D,E). In addition, we also evaluated the function of Lnc18q22.2 on cell migration upon hypoxia condition, it was found that Lnc18q22.2 knockdown attenuated HCC cell migration ability (Figure 2F,G). Our results suggest that Lnc18q22.2 may play an oncogene role in HCC progression under hypoxia condition.

### 3.3 KLF4 transcriptionally regulates Lnc18q22.2 expression

**FIGURE 3:**
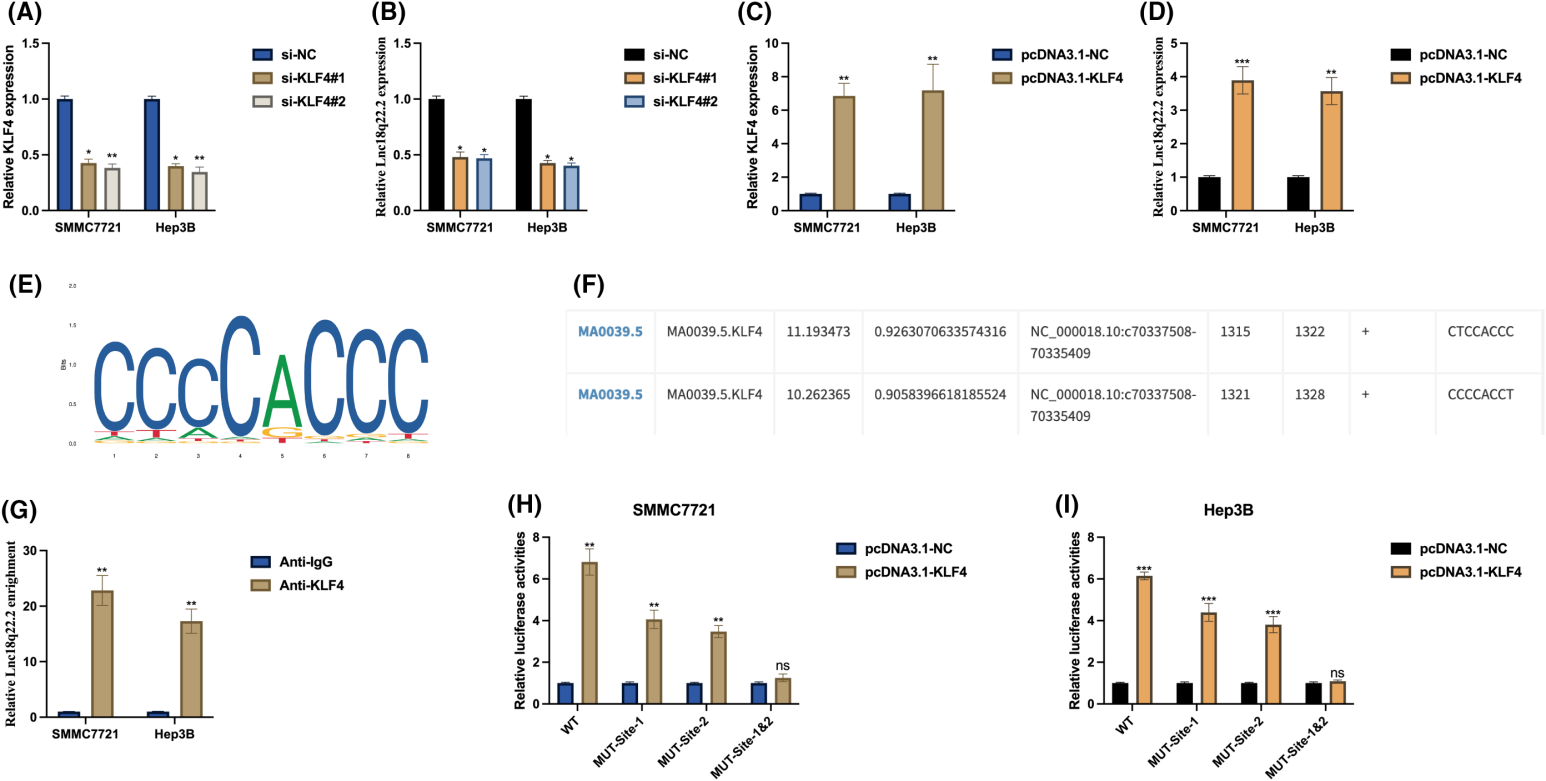
KLF4 transcriptionally regulates Lnc18q22.2 expression. The putative upstream regulator of Lnc18q22.2 was predicted using the JASPAR database (http://jaspar.genereg.net/). (A, B) SMMC7721 and Hep3B cells were stably transfected with si‐NC, si‐KLF4#1, and si‐KLF4#2, the expression of Lnc18q22.2 was measured by qRT‐PCR. (C, D) In SMMC7721 and Hep3B cells, transfection with pcDNA3.1‐KLF4 and its normal control was performed, and Lnc18q22.2 expression was assessed by qRT‐PCR. (E) The binding motif of KLF4 predicted by JASPAR is shown. (F) putative binding sites for the Lnc18q22.2 promoter were determined using JASPAR. (G). The binding possibility between KLF4 and the Lnc18q22.2 promoter was evaluated by ChIP assay. (H, I) The interaction between KLF4 and the Lnc18q22.2 promoter in SMMC7721 and Hep3B cells was verified using the luciferase reporter gene assay. The results were presented as mean ± SD with a sample size of *n* = 3.

To understand the molecular mechanisms underlying the function of Lnc18q22.2 in hypoxia‐induced HCC progression, we investigated the upstream regulator of Lnc18q22.2. Utilizing the JASPAR database, we identified KLF4 as a potential transcription factor (TF) of Lnc18q22.2. Knockdown (Figure 3A,B) and overexpression (Figure 3C,D) of KLF4 in HCC cells revealed that Lnc18q22.2 expression can be positively regulated by KLF4. We hypothesized that KLF4 regulates Lnc18q22.2 expression by acting as a TF. Putative binding sites of KLF4 (Figure 3E) and Lnc18q22.2 (Figure 3F) were obtained from the JASPAR database. ChiP assay results demonstrated that Lnc18q22.2 was highly enriched in KLF4 antibody compared with IgG antibody in SMMC7721 and Hep3B cells (Figure 3G), indirectly supporting the association between KLF4 and Lnc18q22.2. This association was further validated by performing a luciferase reporter gene assay, revealing two active binding sites between KLF4 and Lnc18q22.2 (Figure 3H,I). These results indicate that Lnc18q22.2 expression in HCC cells is transcriptionally regulated by KLF4.

### 3.4 KLF4 modulates HCC tumorigenic properties under hypoxia via Lnc18q22.2

**FIGURE 4:**
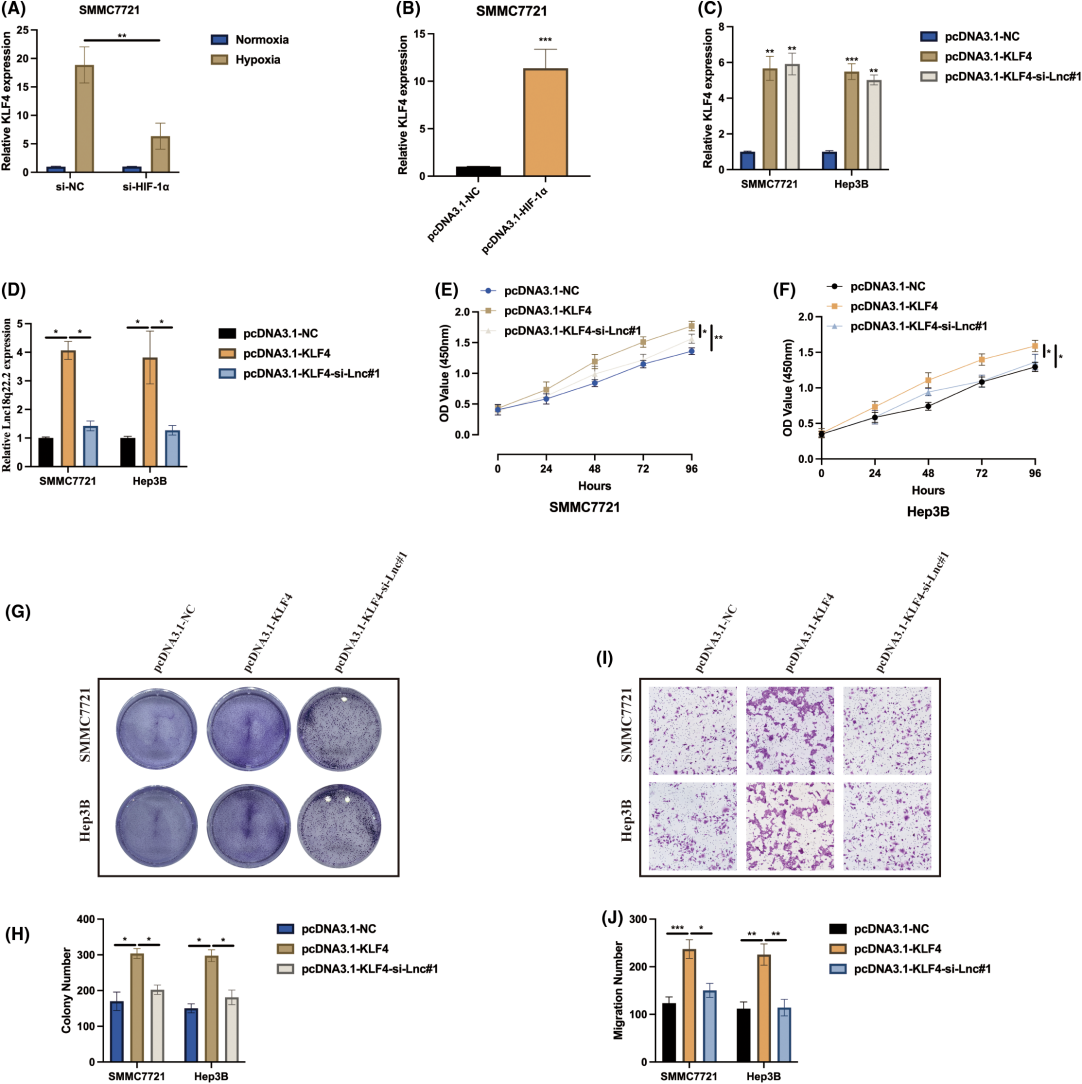
KLF4 modulates HCC tumorigenic properties under hypoxia via Lnc18q22.2. (A, B) SMMC7721 cells underwent transfection with si‐NC, si‐HIF‐1α, pcDNA3.1‐NC and pcDNA3.1‐HIF‐1α. Following a 24‐h transfection period, cells were cultured under either normoxic or hypoxic conditions for an additional 24 h. The expression levels of KLF4 were assessed through qRT‐PCR. (C, D) SMMC7721 and Hep3B cells were transfected with pcDNA3.1‐NC, pcDNA3.1‐KLF4 and pcDNA3.1‐KLF4 + si‐Lnc#1. Following a 24‐h transfection period, cells were cultured under either normoxic or hypoxic conditions for an additional 24 h. The levels of KLF4 and Lnc18q22.2 expression were examined using qRT‐PCR. (E, F). Cell proliferation levels were determined by CCK‐8 assay. (G, H) Cell viability was detected by cell colony assay. (I, J) Cell migration ability was assessed by transwell migration assay. The results were presented as mean ± SD with a sample size of *n* = 3.

Subsequently, the function of KLF4 in hypoxia‐induced HCC was investigated. Initially, it was observed that KLF4 expression can be positively upregulated in hypoxia‐induced HCC cells by HIF‐1α in SMMC7721 cells (Figure 4A,B). Cell models for subsequent experiments were generated by transfecting pcDNA3.1‐NC, pcDNA3.1‐KLF4, pcDNA3.1‐KLF4 + si‐Lnc#1 into HCC cells, and the transfection efficiencies were validated as indicated in Figure 4C,D. As revealed by CCK‐8 (Figure 4E,F) and cell colony (Figure 4G,H) assays, KLF4 overexpression promoted hypoxia‐induced HCC proliferation, and this effect was counteracted by Lnc18q22.2 knockdown. Furthermore, Lnc18q22.2 knockdown also rescued the promotive effect of KLF4 on hypoxia‐induced HCC migration ability, as shown by the transwell migration assay (Figure 4I,J). These results suggest that KLF4 also plays an oncogenic role in HCC progression through regulating Lnc18q22.2 expression under hypoxic conditions.

### 3.5 Lnc18q22.2 interacts and stabilizes ULBP3

**FIGURE 5:**
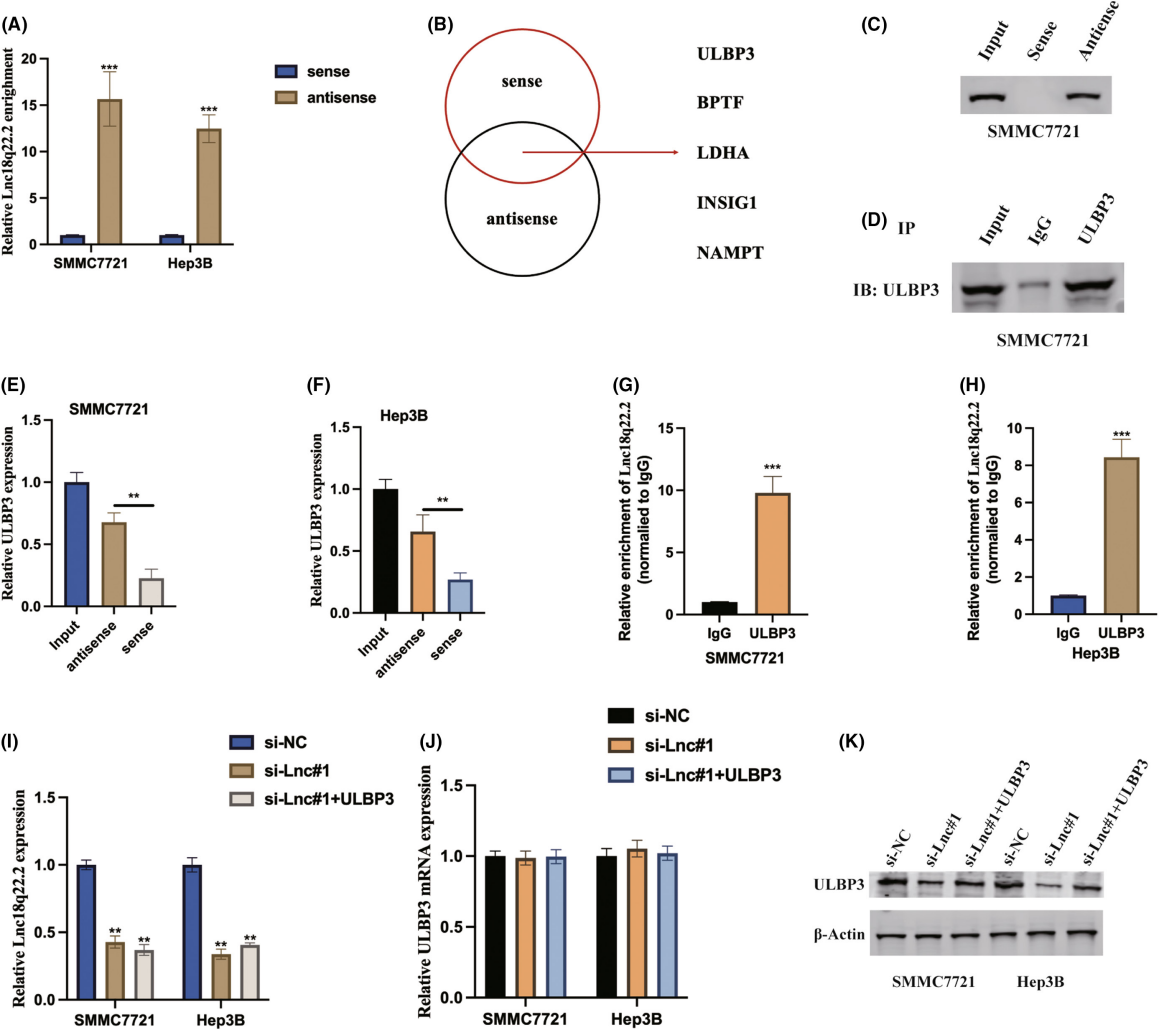
Lnc18q22.2 interacts and stabilizes ULBP3. (A) SMMC7721 and Hep3B cells underwent transfection with both sense and antisense probes designed specifically for Lnc18q22.2. The sense probe corresponds to a DNA probe identical to the Lnc18q22.2 sequence, spanning the back‐splicing junction site, whereas the antisense probe is complementary to Lnc18q22.2. Quantification of Lnc18q22.2 expression was performed using qRT‐PCR, providing a precise measurement of Lnc18q22.2 levels in response to the sense and antisense probes. (B) RNA pull‐down assays coupled with LC–MS/MS mass spectrometry revealed the top five most significantly upregulated putative associated proteins. (C–F) RNA‐protein pulldown assays demonstrated the interaction between Lnc18q22.2 and ULBP3 in SMMC7721 and Hep3B cells. (G, H) RIP and qRT‐PCR assays utilizing ULBP3‐specific antibodies further confirmed the interaction between Lnc18q22.2 and ULBP3. (I) qRT‐PCR assay was performed to measure the expression levels of Lnc18q22.2. (J) Another qRT‐PCR assay was conducted to measure the mRNA expression of ULBP3. (K) Western blot assay was applied to detect the protein expression of ULBP4. The results were presented as mean ± SD with a sample size of *n* = 3.

Expanding our investigation to unravel the intricate molecular mechanisms governed by Lnc18q22.2, we initiated stable transfections of the sense and antisense forms of Lnc18q22.2 into HCC cells, subsequently assessing Lnc18q22.2 expression through qRT‐PCR (Figure 5A). In pursuit of potential protein targets, RNA pull‐down assays followed by liquid chromatography–tandem mass spectrometry (LC–MS/MS) identified ULBP3 as a promising interacting partner of Lnc18q22.2 (Figure 5B). Further substantiating the association between Lnc18q22.2 and ULBP3, immunoblot assays were conducted following pull‐down by the antisense probe (Figure 5C–F), elucidating the predominant role of Lnc18q22.2 as a ULBP3‐associating factor. This interaction was further validated within cellular contexts through RNA immunoprecipitation (RIP) assays utilizing an anti‐ULBP3 antibody (Figure 5G,H). These collective findings shed light on the intricate interplay between Lnc18q22.2 and ULBP3. Furthermore, our results found that Lnc18q22.2 regulated ULBP3 expression at post‐transcription level, Lnc18q22.2 upregulated ULBP3 protein expression but not ULBP3 protein expression in HCC cells (Figure 5I,K).

### 3.6 ULBP3 regulates HCC tumorigenic properties and immune escape under hypoxia

**FIGURE 6:**
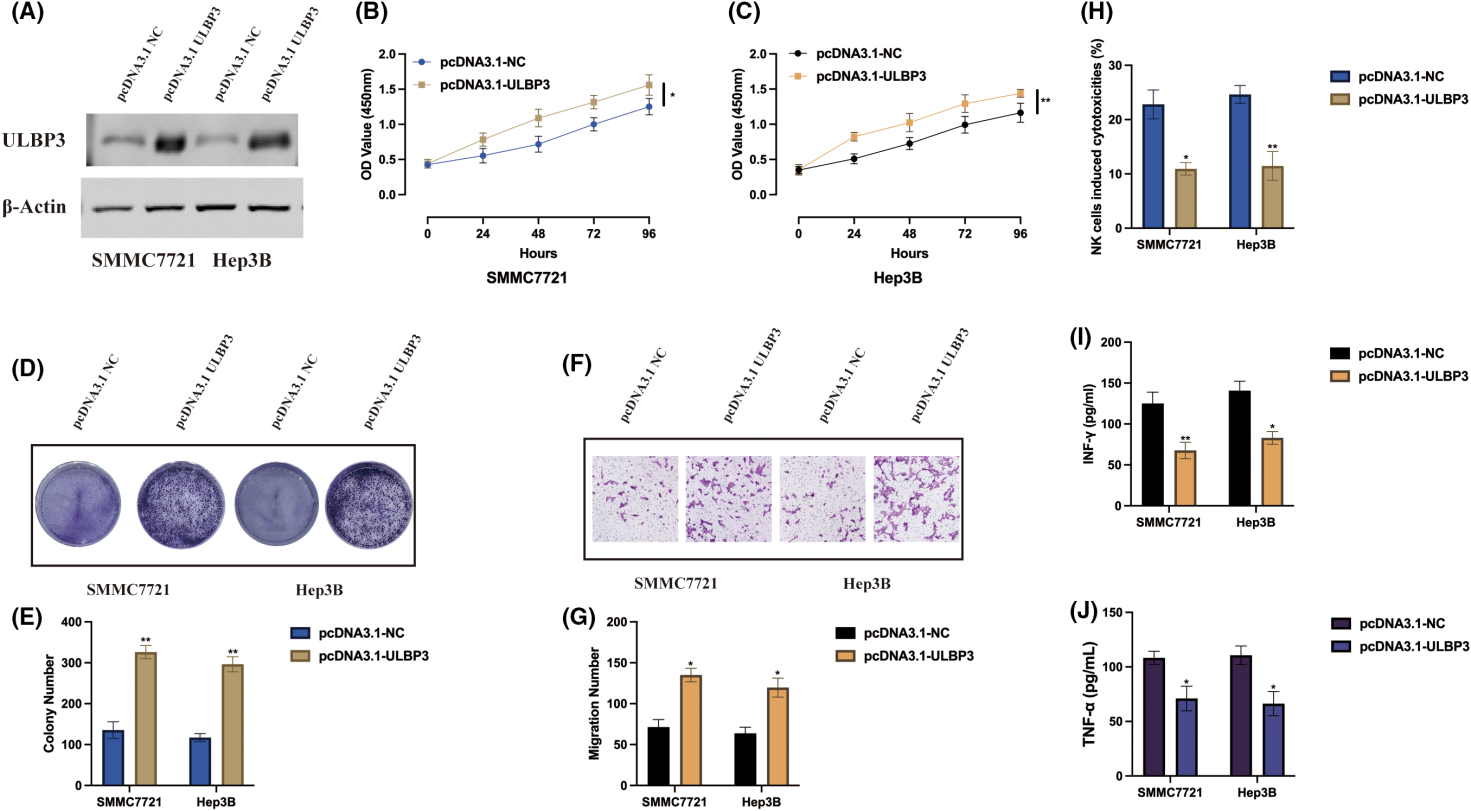
ULBP3 regulates HCC tumorigenic properties and immune escape under hypoxia. (A) SMMC7721 and Hep3B cells underwent transfection with pcDNA3.1‐NC, and pcDNA3.1‐UBLP3. Following a 24‐h transfection period, cells were cultured under either normoxic or hypoxic conditions for an additional 24 h. The expression levels of UBLP3 were assessed through western blot assay. (B, C) Cell proliferation levels were determined by CCK‐8 assay. (D, E) Cell viability was detected by cell colony assay. (F, G) Cell migration ability was assessed by transwell migration assay. (H) Cytotoxicity of NK92 cells was assessed using the CytoTox96 cytotoxicity assay. (I, J) The levels of secreted IFN‐γ and TNF‐α in the cell culture supernatant of the co‐culture system were measured using ELISA assay. The results were presented as mean ± SD with a sample size of *n* = 3.

Due to the function of ULBP3 in hypoxia induced HCC progression remains unclear, we generated ULBP3 overexpression cell models upon hypoxia condition, transfection efficiencies were also validated by western blot (Figure 6A). ULBP3 promoted HCC cell proliferation level upon hypoxia condition was validated by CCK‐8 (Figure 6B,C) and cell colony (Figure 6D,E) assays. Additionally, we also assessed cell migration ability upon ULBP3 overexpression, it was found ULBP3 aggravated HCC cell migration level upon hypoxia condition (Figure 6F,G). As demonstrated by a previous study, ULBP3 can influence immune escape phenomena.^17^ Thereafter, we also examined the impact of ULBP3 on the cytotoxicity of NK cells, we employed IL‐2‐stimulated HCC cells. Co‐culturing NK92 cells with ULBP3 overexpression cells revealed noteworthy results. ULBP3 overexpression in HCC cells not only attenuated NK92 cell cytotoxicity (Figure 6H) but also decreased the secretion of IFN‐g and TNF‐a (Figure 6I,J). The observed trends suggest that ULBP3 may function as an oncogene, suppressing NK92 cell cytotoxicity against hypoxia induced HCC cells.

### 3.7 Identify the role of KLF4/Lnc18q22.2/ULBP3 axis in HCC progression under hypoxia

**FIGURE 7:**
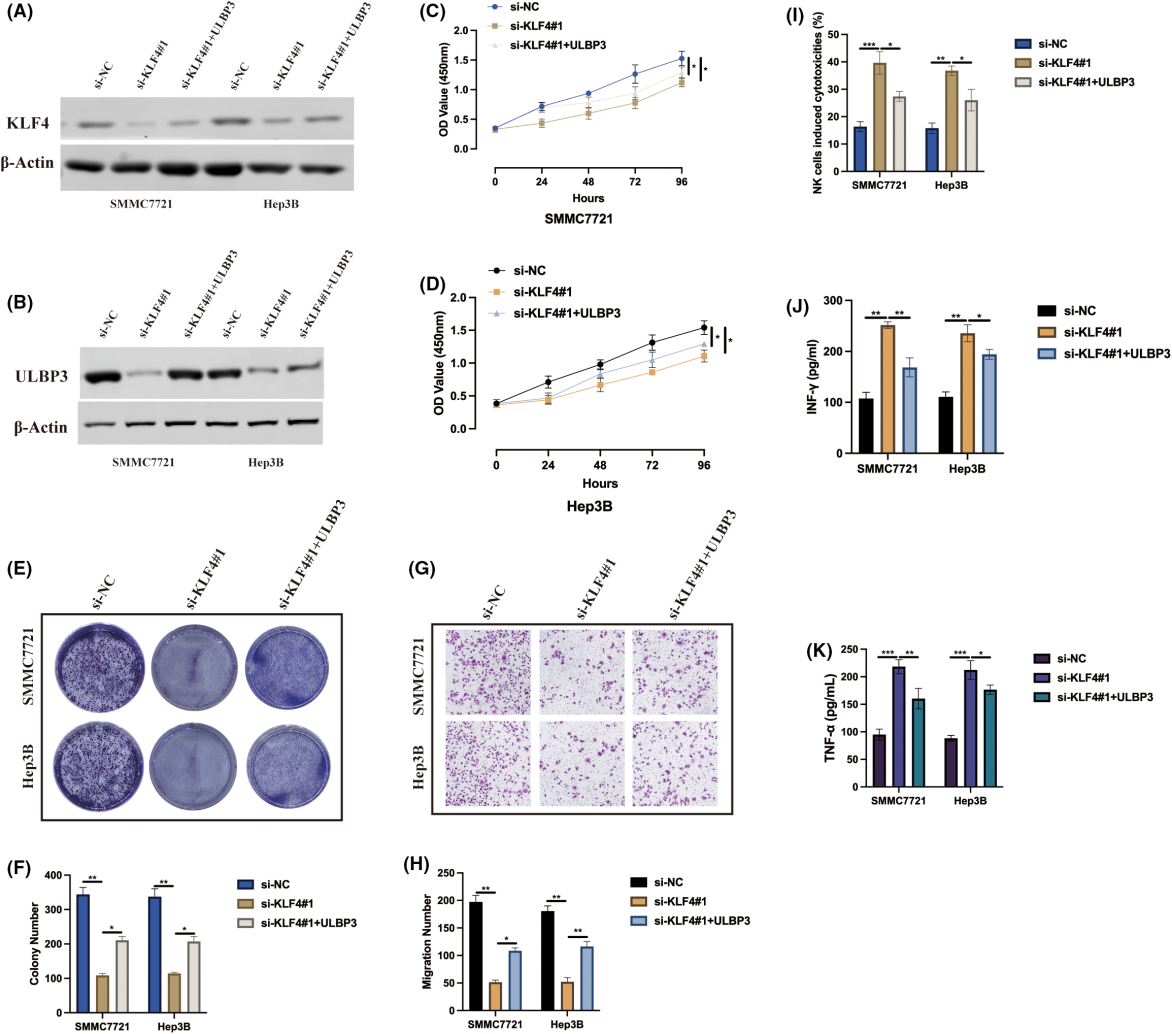
Identify the role of KLF4/Lnc18q22.2/ULBP3 axis in HCC progression under hypoxia. (A, B). SMMC7721 and Hep3B cells underwent transfection with si‐NC, si‐KLF4#1, and si‐KLF4#1 + UBLP3. Following a 24‐h transfection period, cells were cultured under either normoxic or hypoxic conditions for an additional 24 h. The expression levels of KLF4 and UBLP3 were assessed through western blot assay. (C, D) Cell proliferation levels were determined by CCK‐8 assay. (E, F) Cell viability was detected by cell colony assay. (G, H) Cell migration ability was assessed by transwell migration assay. (J) Cytotoxicity of NK92 cells was assessed using the CytoTox96 cytotoxicity assay. (J, K) The levels of secreted IFN‐γ and TNF‐α in the cell culture supernatant of the co‐culture system were measured using ELISA assay. The results were presented as mean ± SD with a sample size of *n* = 3.

Taken together, we assessed our findings, a novel KLF4/Lnc18q22.2/ULBP3 axis, in HCC progression under hypoxia condition by transfecting si‐NC, si‐KLF4#1, si‐KLF4#1+ULBP3 into HCC cells and then subjected to cellular experiments upon hypoxia condition. Transfection efficiencies were measured by western blot as indicated in Figure 7A,B. As revealed by CCK‐8 (Figure 7C,D) and cell colony (Figure 4E,F) assays, KLF4 knockdown inhibited hypoxia‐induced HCC proliferation, and this effect was counteracted by ULBP3 overexpression. ULBP3 overexpression also rescued the inhibitive effect of KLF4 knockdown on hypoxia‐induced HCC migration ability, as shown by the transwell migration assay (Figure 7G,H). Furthermore, KLF4 knockdown in HCC cells aggravated NK92 cell cytotoxicity (Figure 7I) but also increased the secretion of IFN‐g and TNF‐a (Figure 7J,K), and those effects were aborted by ULBP3 overexpression. These results suggest that KLF4 plays an oncogenic role in HCC progression through regulating Lnc18q22.2/ULBP3 axis under hypoxic conditions.

## 4 DISCUSSION

LncRNAs are RNA molecules with a transcript length exceeding 200 nucleotides and do not possess protein‐coding abilities. Significantly, lncRNAs have emerged as crucial regulators of gene expression, playing key roles in a wide array of biological processes and being implicated in various diseases, including cancer.^18^, ^19^ Recent investigations have uncovered that lncRNAs play integral roles not only in the proliferation, apoptosis, migration and invasion of numerous malignant tumours but also in other critical cellular processes.^20^ Furthermore, it has been demonstrated that hypoxia‐induced lncRNAs play a role in promoting tumour metastasis.^21^ In recent years, there has been a growing focus on understanding the interplay between hypoxia, lncRNAs and cancer. For example, hypoxia induced TUFT1 is suggested to enhance HCC growth and metastasis by activating the Ca^2+^/PI3K/AKT pathway.^22^ Another study uncovered that hypoxia‐induced HMGB1 mediates HCC tumour growth through Toll‐like receptor 9.^23^ In this study, our goal was to identify a novel long noncoding RNA (lncRNA) exhibiting dysregulated expression in hypoxic HCC cells, aiming to provide new insights into the potential regulatory mechanisms of hypoxia‐induced lncRNAs in HCC. By utilizing lncRNA microarray analysis on hypoxia induced HCC cells, we found that Lnc18q22.2 was the most significantly upregulated lncRNA. Cellular experiments verified that Lnc18q22.2 played an oncogene role in hypoxia induced HCC progression through promoting HCC cell proliferation and migration level upon hypoxia condition.

Currently, there is little known about the biological role also with molecular function of Lnc18q22.2. Due to the molecular mechanisms underlying transcription factor regulating lncRNA expression have been well documented in the past decade,^24^, ^25^, ^26^, ^27^ In light of the observed upregulation of Lnc18q22.2 in HCC cells under hypoxic conditions, our research endeavoured to ascertain whether this trend could be attributed to modulation by a transcription factor. Our findings reveal that the transcription factor KLF4 plays a pivotal role in the regulation of Lnc18q22.2 expression within HCC cells. Moreover, KLF4 was found to enhance both the proliferation and migration of HCC cells under hypoxic conditions by modulating Lnc18q22.2 expression. This discovery offers a novel perspective on the function of KLF4 in HCC progression, suggesting a previously unappreciated direction for interpreting the role of this transcription factor in the disease's development.

Our investigation has elucidated the intricate relationship between Lnc18q22.2 and its downstream effector, ULBP3, within the context of HCC cells. This relationship is particularly pronounced in the stabilization of ULBP3 expression, a protein previously associated with immune evasion yet possessing an ambiguous role in HCC pathogenesis. Through rigorous experimentation, we have delineated the biological functions of ULBP3 in the advancement of hypoxia‐induced HCC, demonstrating its capacity to enhance cell proliferation and migration, while concurrently attenuating immune escape mechanisms under hypoxic conditions.

Critically, our research underscores the pivotal role of KLF4 in HCC progression, facilitated by its regulation of the Lnc18q22.2/ULBP3 axis under hypoxic stress. These insights not only advance our comprehension of the KLF4/Lnc18q22.2/ULBP3 axis's influence on hypoxia‐induced HCC tumorigenesis, particularly regarding immune evasion, but also underscore the potential of this axis as a therapeutic target.

In summary, the discovery of the KLF4/Lnc18q22.2/ULBP3 axis marks a significant contribution to our understanding of the mechanisms underlying hypoxia‐induced HCC progression. The implications of this finding are profound, offering promising new directions for the development of targeted therapeutic interventions aimed at combating HCC. This novel axis represents a pivotal step forward in our quest to delineate the molecular intricacies of HCC, holding substantial potential for enhancing patient outcomes through the identification of innovative treatment strategies.

## AUTHOR CONTRIBUTIONS

**Lifang Wei:** Data curation (equal); formal analysis (equal); investigation (equal); software (equal); visualization (equal). **Ping He:** Data curation (equal); formal analysis (equal); methodology (equal); software (equal); visualization (equal). **Zhongqiu Tan:** Formal analysis (equal); software (equal). **Lifeng Zhao:** Data curation (equal); software (equal). **Cheng Lin:** Conceptualization (equal); data curation (equal); investigation (equal); methodology (equal); project administration (equal); resources (equal); supervision (equal); writing – original draft (equal). **Zhongheng Wei:** Conceptualization (equal); investigation (equal); methodology (equal); project administration (equal); supervision (equal); writing – original draft (equal).

## FUNDING INFORMATION

This work was supported by the National Nature Science Foundation of China (No. 82260532).

## CONFLICT OF INTEREST STATEMENT

The authors have declared that no competing interest exists.

## Data Availability

The data that support the findings of this study are available on request from the corresponding author.
